# Gene Network Homology in Prokaryotes Using a Similarity Search Approach: Queries of Quorum Sensing Signal Transduction

**DOI:** 10.1371/journal.pcbi.1002637

**Published:** 2012-08-16

**Authors:** David N. Quan, William E. Bentley

**Affiliations:** 1 Fischell Department of Bioengineering, University of Maryland College Park, College Park, Maryland, United States of America; 2 Institute for Bioscience and Biotechnology Research, College Park, Maryland, United States of America; University of Illinois at Urbana-Champaign, United States of America

## Abstract

Bacterial cell-cell communication is mediated by small signaling molecules known as autoinducers. Importantly, autoinducer-2 (AI-2) is synthesized via the enzyme LuxS in over 80 species, some of which mediate their pathogenicity by recognizing and transducing this signal in a cell density dependent manner. AI-2 mediated phenotypes are not well understood however, as the means for signal transduction appears varied among species, while AI-2 synthesis processes appear conserved. Approaches to reveal the recognition pathways of AI-2 will shed light on pathogenicity as we believe recognition of the signal is likely as important, if not more, than the signal synthesis. LMNAST (Local Modular Network Alignment Similarity Tool) uses a local similarity search heuristic to study gene order, generating homology hits for the genomic arrangement of a query gene sequence. We develop and apply this tool for the *E. coli lac* and LuxS regulated (Lsr) systems. Lsr is of great interest as it mediates AI-2 uptake and processing. Both test searches generated results that were subsequently analyzed through a number of different lenses, each with its own level of granularity, from a binary phylogenetic representation down to trackback plots that preserve genomic organizational information. Through a survey of these results, we demonstrate the identification of orthologs, paralogs, hitchhiking genes, gene loss, gene rearrangement within an operon context, and also horizontal gene transfer (HGT). We found a variety of operon structures that are consistent with our hypothesis that the signal can be perceived and transduced by homologous protein complexes, while their regulation may be key to defining subsequent phenotypic behavior.

## Author Summary

Bacteria communicate with each other through a network of small molecules that are secreted and perceived by nearest neighbors. In a process known as quorum sensing, bacteria communicate their cell density and certain behaviors emerge wherein the population of cells acts as a coordinated community. One small signaling molecule, AI-2, is synthesized by many bacteria so that in a natural ecosystem comprised of many secreting cells of different species, the molecule may be present in an appreciable concentration. The perception of the signal may be key to unlocking its importance, as some cells may recognize it at lower concentrations than others, etc. We have created a searching algorithm that finds similar gene sets among various bacteria. Here, we looked for signal transduction pathways similar to the one studied in *E. coli*. We found exact replicas to that of *E. coli*, but also found pathways with missing genes, added genes of unknown function, as well as different patterns by which the genes may be regulated. We suspect these attributes may play a significant role in determining quorum sensing behaviors. This, in turn, may lead to new discoveries for controlling groups of bacteria and possibly reducing the prevalence of infectious disease.

## Introduction

Comparing prokaryotic whole genome sequences to identify operons is a mature area of research [1], [2], [3], [4]. Orthologous operon identification can imply a secondary degree of relation between components, reaffirming Clusters of Orthologous Groups (COG) and other assignments of function as well as suggesting essentiality [5]. This conservation of components also speaks to the conservation of signaling capacity in orthologous modular signaling operon-based units. That is, we are interested in ascertaining the genetic modularity of signal transduction processing, in particular those that operate within known, putative regulons. Drawing partly on previous work investigating microsynteny and gene neighborhoods [3], [6], [7], we developed a general similarity search approach, we call a Local Modular Network Alignment Similarity Tool (LMNAST). LMNAST applies a BLAST-like heuristic to gene order and arrangement. Resultant search hits help capture the conservation and phylogenetic dispersion of a given query modular network.

Using, as queries, contiguously abutting genes of prokaryotic modular signaling networks, LMNAST identifies and scores hits based on the minimum number of frank mutations in gene organization needed to arrive at a given putative system homolog when starting from the query. Here, homology refers to similarity in relative gene order and relative transcriptional direction, after nucleotide level threshold filtering of gene elements based on BLAST [8] E-value.

**Figure 1:**
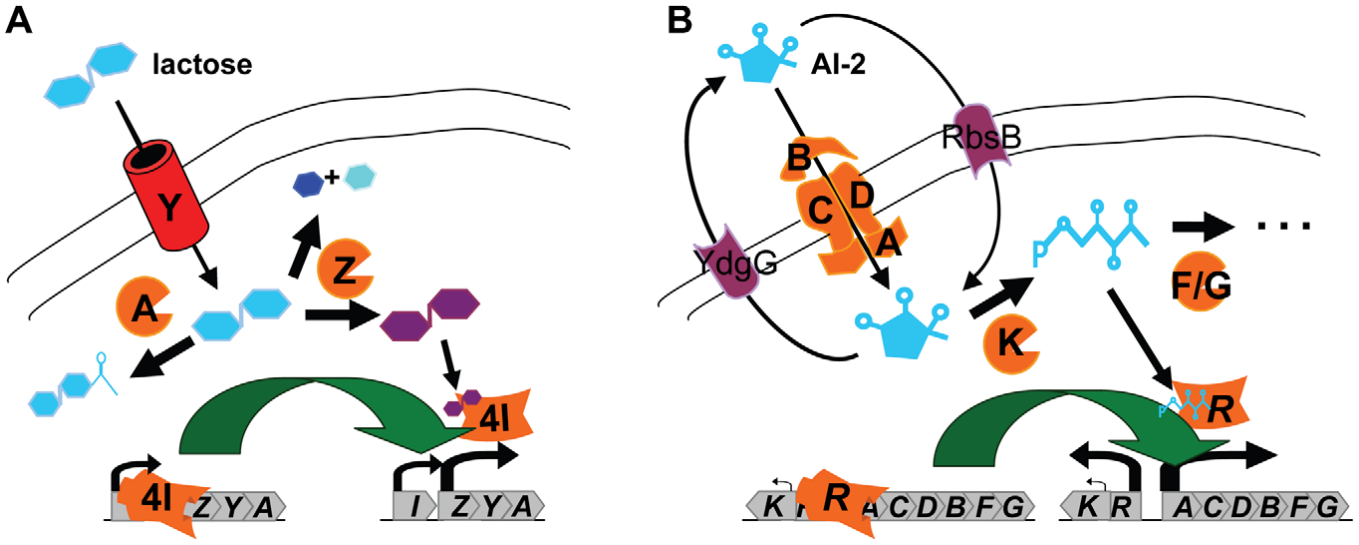
Test queries: *lac* operon and Lsr system. A. The *lac* operon is composed of beta-galactosidase (LacZ), the lactose importer (LacY), and a beta-galactoside transacetylase (LacA). Upstream of the operon, the operon repressor (LacI) is expressed in a co-directional orientation. The primary function of the *lac* system is as a regulated importer/processing unit. Lactose brought in through the permease LacY is converted into allolactose or hydrolyzed into glucose and beta-galactose. Both reactions are catalyzed by LacZ. Allolactose then acts to release the repression of the system by LacI. B. The Lsr system is composed of two divergent operons. One operon consists of an AI-2 kinase and a system repressor. The other operon consists of an AI-2 transporter and phospho-AI2 processing genes. Contextual system behavior is partly governed by distinctly regulated parts including an alternative importer [23], an exporter [33], and the AI-2 synthase gene. Relative to the canonical *lac* system, the Lsr system is complicated by the fact that the cell synthesizes,exports, and imports AI-2, and by the negative regulation associated with the divergently arranged structure. AI-2 exported by a mechanism involving YdgG traverses the outer membrane through a porin and enters the periplasmic space. Through the ABC-type importer, LsrACDB, AI-2 is then transported back into the cytosol. Once there, AI-2 is phosphorylated by LsrK. This phosphorylated form (AI-2-P) derepresses the Lsr system and is catabolized by LsrF and LsrG into separate downstream products.

For the purpose of evaluation, two small modular systems were used as test inputs: one was the *E. coli lac* system and the other was the LuxS regulated (Lsr) system. In some ways, the two systems are quite similar (Fig. 1). Both import and catabolize the small molecules that induce system expression. For the *lac* system, this small molecule is, of course, lactose. For the Lsr system, the small molecule is autoinducer-2 (AI-2). AI-2 is a signaling molecule common among at least eighty bacterial species [9]. As mediated through either the Lsr or LuxPQ systems, bacteria are believed to use AI-2 to guide population based phenotypes, a phenomenon termed quorum sensing [10]. LuxPQ is a histidine kinase two component system, the regulon of which is distinct from Lsr and is not considered further. Lsr is an interesting query because its distribution should help elucidate its putative, modular quorum sensing function [9] and because the known homologs differ in gene organization [10], [11], [12].

## Methods

**Figure 2:**
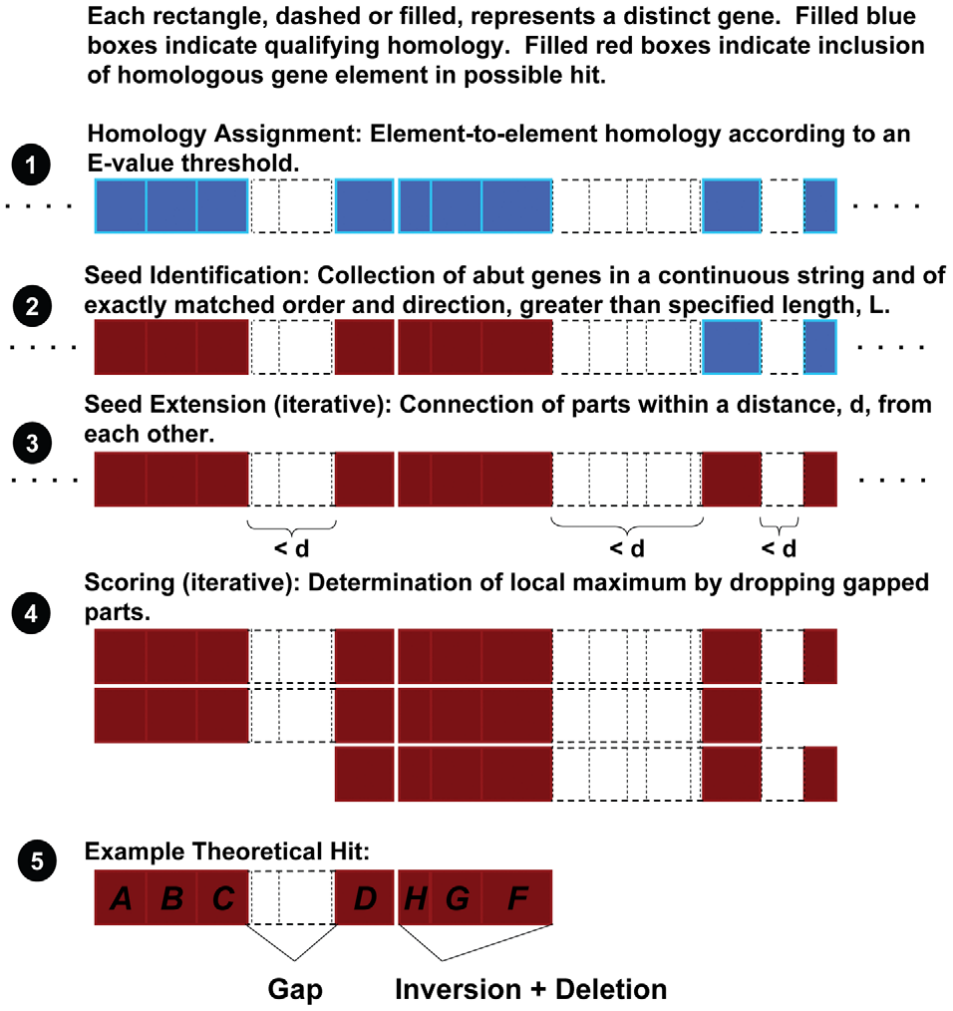
LMNAST heuristic. LMNAST operates in a BLAST-like manner, using the results of BLAST searches themselves as a curated database. 1. For each member of the query, in any nucleotide record, a homolog's membership to a character type is assigned by scoring below a specified BLAST E-value threshold. Genes assigned to characters are highlighted blue. Genes without sufficient homology to any character are represented by dashed boxes. 2. Sufficiently long stretches of adjacent characters are identified as seeds (red). 3. Sufficiently proximal characters are connected to seeds or seeds are connected to each other when separated by a base pair distance<d. 4. Rearrangements, losses, and deletions are scored according to a standard similarity heuristic. Noncontinuous elements are dropped iteratively until a maximum score is achieved, arriving at… 5. An LMNAST hit.

As previously indicated, LMNAST evaluates nucleotide records for similarity to a query network using a BLAST-like heuristic. Specifically, it treats the gene order of query networks as a string of characters. Queries are therefore not necessarily restricted to defined networks insofar as any gene ordering may be a query. A standard heuristic of penalties for various rearrangements of orthologous systems is employed. For searches described herein, a loose threshold was used to generate an exhaustive set of hits. The overall scheme is depicted in Fig. 2. The program itself is available at http://www.bentley.umd.edu.

### Input

Input consisted of an ordered list of gene elements (for example, *lacIZYA*). For each gene element a BLAST result file was generated using tblastn to search the nr/nt database for hits with E-values less than 0.1, narrowing the search space. Each BLAST hit was assigned a character corresponding to the gene element queried. BioPerl [13] was used to query Genbank databases and process data from retrieved files. Nucleotide records with sufficiently proximal characters were investigated further.

### Scoring Heuristic

The degree of similarity between a putative hit and its corresponding query was evaluated according to the number of deletions, insertions, and rearrangements required to generate the putative hit from the query. Intra-hit gene duplications were disallowed as a simplification. Consequently, deletion could be noted by character type inclusion. Insertions of uncharactered elements between gene homologs were scored according to an affine gap rule whereby a portion of the deduction was scaled to the insertion length. Rearrangements refer to altered relative order and relative gene direction. Changed relative direction was only considered when relative order was maintained. When this criterion was satisfied, relative order was evaluated in terms of adjacent homolog distance, disregarding insertions and deletions. For each such structural dissimilarity there was a standard deduction in score. Noncontiguous elements were dropped iteratively until a maximum score was reached for each putative hit. When more than one putative hit version elicited equal scores in the same round, the version of the hit with the most characters was favored. Putative hits with scores greater than zero were retained.

### Weak and Stringent Criteria

For evaluation purposes and to find a suitable balance between false positives and coverage completeness, each test query was run under both weak and stringent conditions. Stringent criteria searches assumed accurate annotation. Contrarily, weak criteria did not require genes to lie within the annotated coding sequence. Moreover, characters annotated as “pseudo” or bounded outside “gene” annotation were accepted as homologous characters. Weak criteria searches also allowed multiple genes to co-exist within the same annotation. Additionally, as a concession to the possibility of longer range interactions between genes, reduced gap penalties were used in weak criteria searches. Results described herein were derived using a gap penalty of 1 and 2 with an extension penalty of 0.3 and 1, for weak and stringent criteria searches respectively.

### Ancillary LMNAST Search Tools

Mean element homology (meH) is a normalized, ancillary measure of string similarity as evaluated by BLAST. Useful for contrasting BLAST results to LMNAST hits, meH was calculated by normalizing each gene homolog's bit score to the maximum bit score for the entire corresponding BLAST result given a background subtraction of the minimum bit score. These normalized bit scores were then averaged for all gene elements within an LMNAST hit. A score of one indicates exact likeness whereas zero indicates the least degree of similarity.

Also, widening the query beyond the system of interest to include a nominal number of flanking genes, here termed “extended window searching,” afforded additional contextualization of LMNAST hit results.

Finally, in evaluating certain low homology hits, nonscoring synonyms were used. Nonscoring synonyms are elements with equivalent gene annotation but insufficient homology according to the initial E-value filter. This is somewhat analogous to replacement in blastp.

## Results

### *E. coli* K-12 W3110 *lac* Operon Query

**Figure 3:**
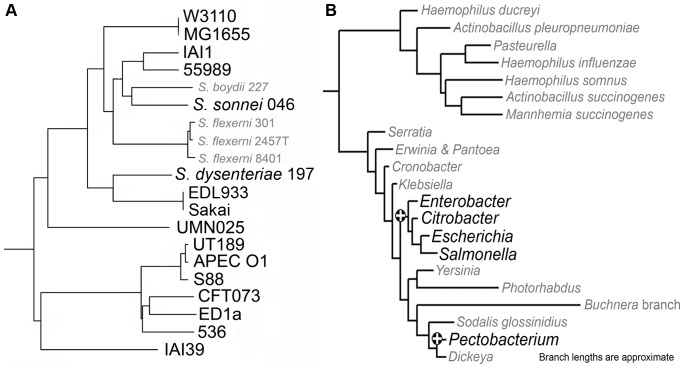
Lac operon LMNAST hits overlaid onto phylogenetic distributions of different scopes. The larger, bolded leaves represent species that contain *lac* operon homologs, whereas the grayed italicized leaves were completely bereft. A. An *E. coli* specific phylogenetic tree as adapted from [4], wherein all genes from the core *E. coli* were used to construct a consensus tree. Among the species/strains represented here, *lac* system homologs were absent from certain *Shigella*. Additionally, BW2952, SE11, IAI1, HS, E243227A, CFT073, K-12 DH10B, and 11 of 18 O:H serotyped strains contained truncated systems. Uniquely, CFT073 retains *lacA*, while missing *lacI* in an otherwise preserved *lac* system structure. B. The phylogenetic dispersion of the *lac* system is mostly limited to *Escherichia* and proximal species, as seen in the 16s based tree adapted from [5]. Bolded leaves indicate the presence of the *lac* system in at least one strain.

We began evaluation of LMNAST by searching for the well characterized *E. coli lac* operon. Specifically, the *E. coli lac* genes *lacI* (BAE76127), *lacZ* (BAE76126), *lacY* (BAE76125), and *lacA* (BAE76124) (spanning bp 360473 to 366734 of the Genbank nucleotide record AP009048) were used as a query. The stringent criteria search yielded fewer hits than the corresponding weak criteria search (189 vs. 236). Of the hits derived from the stringent criteria search, complete and perfectly arranged *lac* systems were found in 26 unique *E. coli* strains and *S. enterica* arizonae serovar 62:z4,z23 (meH 0.8), the only *Salmonella enterica* serovar represented among all *lac* system hits, in keeping with its significant divergence from other serovars [14]. A representation of *E. coli* hits in a phylogenetic context is available in Fig. 3a. The average meH (0<meH<1) for these complete systems was 0.98. An extended window query with five additional genes on either side of the original search frame, revealed eight complete systems with a hitchhiking, proximal cytosine deaminase after losing all other proximal genes. Only one system with all four characters was entirely removed from the original query's proximal gene set, suggestive of negligible stability for the canonical system outside of a limited phylogenetic domain.

An additional 28 hits were bereft one *lac* system character (average meH 0.74). In all but three of these cases that missing gene was *lacA*. Of these hits, ten had an additional frank structural change to a divergent expression pattern originating between *lacI* and *lacZ* characters (e.g. in *E. cloacae*), likely increasing system sensitivity to *lacI* repression in these cases [15]. Surprisingly, in other instances, extended window searching revealed the only proximal structural change to be a missing *lacA* gene. This *lacA* degeneracy may be indicative of its relative functional unimportance compared to other *lac* system members [16].

**Figure 4:**
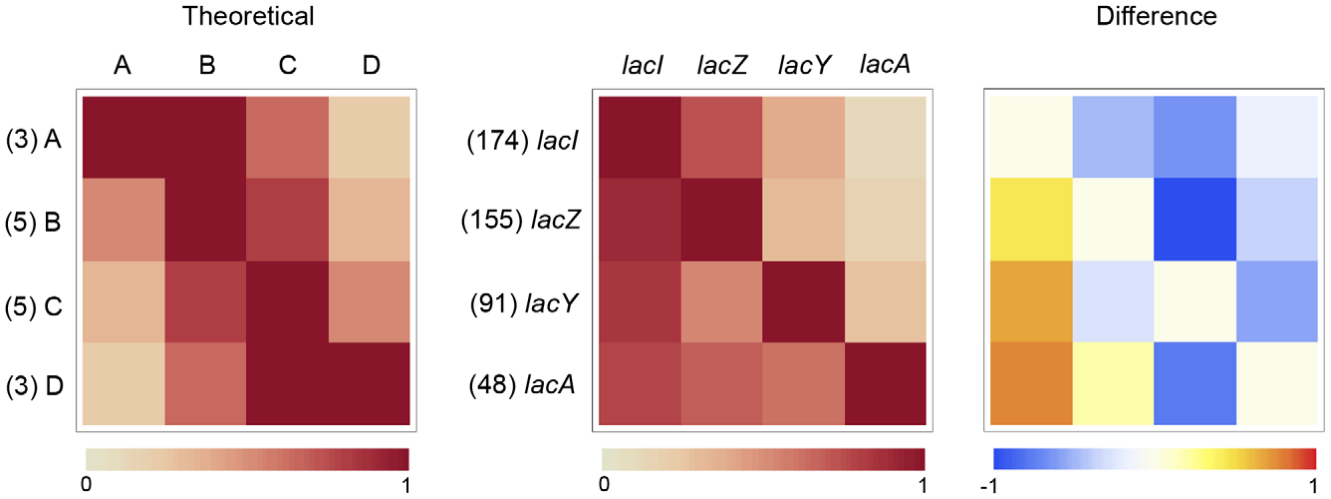
Coincidence heat map for Lac operon LMNAST stringent search hits. Each shaded index represents the normalized frequency of hits containing the row gene that also contain the column gene out of the total number of hits containing the row gene, as denoted by (#). The matrix on the left is a representation of an unbiased set of evenly distributed homologs (AB, BC, CD, ABC, BCD, and ABCD). The middle matrix is the actual coincidence data. The matrix on the right is a heat map of the difference between the two. *LacI* is heavily over-represented according to the difference matrix. *LacY* occurred less than would be expected according to a random distribution.

Some of the patterns described above can be inferred from coincidence heat charts (Fig. 4). These matrices represent LMNAST results by the frequency of coincidence between gene characters within hits. The shade of an index represents the frequency of hits where the row gene coincides with the column gene, normalized against the total number of hits containing the row gene, which itself is denoted by (#). For example, in Fig. 4, the left-most matrix is a representation of a theoretical set of homolog fragments (AB, BC, CD, ABC, BCD, and ABCD). This simple set was constructed to only reflect unbiased homologous recombination presumably resulting only in chromosomal rearrangements. In this set, B and C were extant in five inputs, while A and D were extant in three inputs. All three inputs containing A also contained B, two also contained C, and one also contained D. This is reflected in the shades of the grids in the top row.

The middle matrix represents the coincidence distribution among LMNAST *E. coli lac* hits. As an additional example, matrix element (2,1) is a rust color representing the 139 hits with a *lacI* character of the 155 also containing *lacZ*. Finally, the right-most matrix is the difference between the left and middle matrices. This particular analysis suggests, for example, that *lacI* is relatively over-represented across all hits, and that nearly all other coincidences are under-represented; surprisingly, this includes coincidences involving the permease, *lacY*. Unlike *lacA*, *lacY* is believed necessary for lactose catabolism, possibly pointing to the use of a lower affinity transporter in such cases. On the other hand, the over representation of *lacI* indicates an expected preference for the regulation of lactose catabolism.

Of the strong criteria search results, 138 hits contained only two *lac* gene homologs (average meH 0.28). Two gene homologs represent the natural minimum of individual characters that a homologous system may contain. Such hits represented truncated systems, repurposed individual members, homoplasic convergence, or outright false positives. The majority of these hits fell within clusters of shared Genbank annotation in 2D similarity plots, which compare meHs (averaged BLAST homologies) against LMNAST homologies, or put differently, average amino acid identities against the system's broader organizational identity. Generically then, purely vertical displacements imply perfect conservation across species through either vertical or, more likely, recent horizontal gene transfer accompanied by amelioration, while purely horizontal displacements indicate recent gene loss and/or rearrangement. For purposes of downstream analysis, it is interesting to speculate that the kinetics of the remaining genes are unaffected in cases of purely horizontal displacement. For systems subject to HGT, such liberties must necessarily be taken with less confidence.

**Figure 5:**
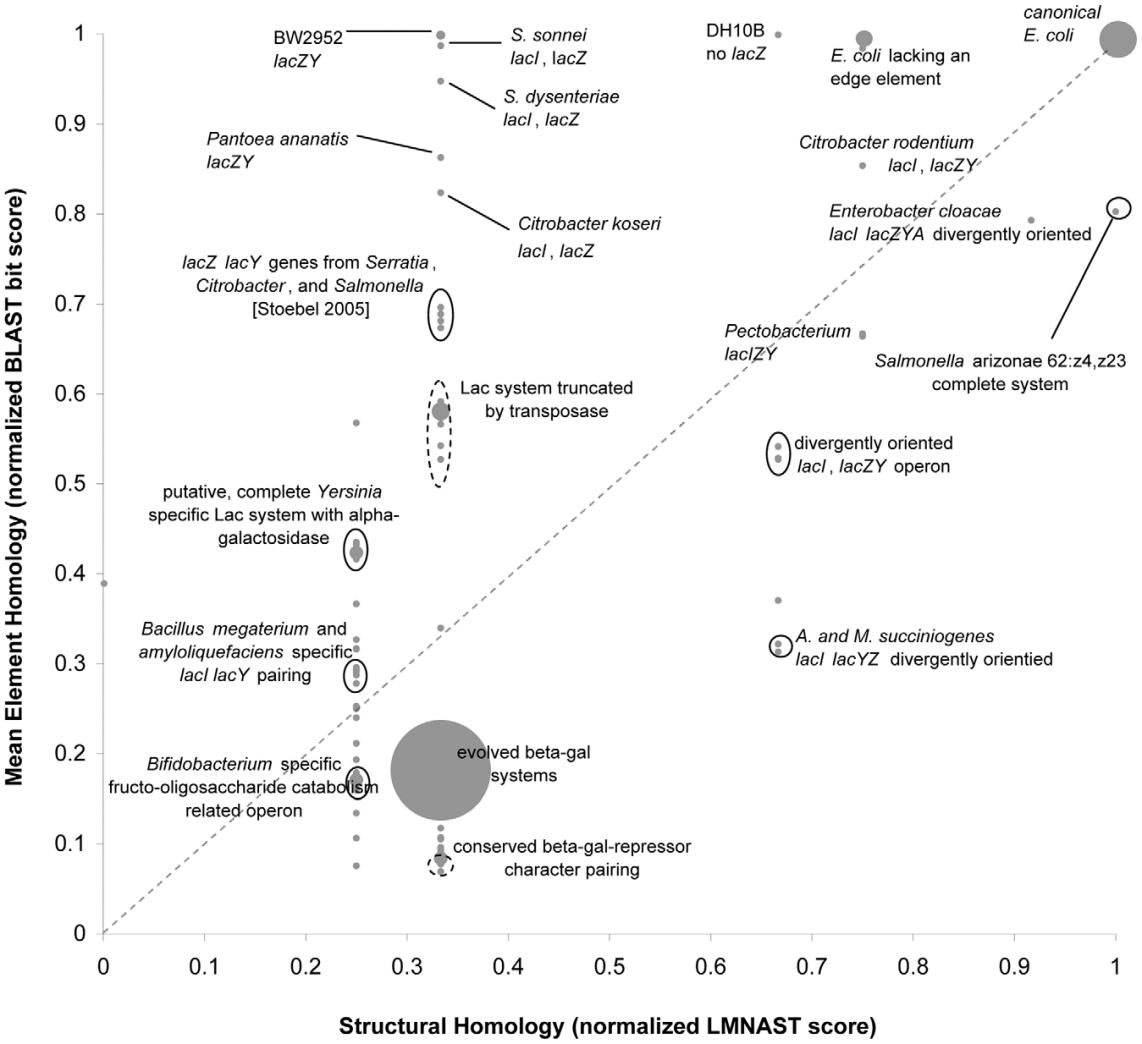
2D Similarity Plot of Lac operon LMNAST stringent search hits overlaid with attributed annotation. Each gray dot represents the homology coordinate of a hit. The size of the dot scales directly with the number of hits at the same coordinate. The dashed line is a 1∶1 line along which hits have the same degree of homology by both BLAST and LMNAST measures. Seemingly vertical displacements may imply horizontal gene transfer, while horizontal displacements may imply gene loss or arrangement within the same or proximal species. Ovals indicate a clustering of similarly annotated hits. Dashed ovals denote cases where only the majority of hits therein share the labeled Genbank annotation. Here, the dominant features are systems mirroring the original structure and the evolved beta-galactosidase system. Very little HGT is apparent while gene loss and rearrangement are ostensibly more common.

In the case of the stringent *lac* search, similarity plots revealed a great deal of structural variability in the *lac* operon homologs of *E. coli* and near *E. coli* species (Fig. 5). Nonetheless, the canonical *lac* operon (26) and the paralogous evolved beta-galactosidase system (43) [17] are clearly the most dominant *lac* operon-homologs, perhaps partially reflecting the relative preponderance of fully sequenced *E. coli* strains.

Addressing the full breadth of two character homologs, 87 contained *lacZ* and *lacY* character types, all of which were adjacent, five of which were misdirected relative to one another. Numerous truncated systems had high meH but imperfect organizational similarity. This cohort was restricted to strains of *E. coli* and closely related *Shigella*, *Citrobacter*, and *Enterobacter* species, reflecting a generally confined phylogenetic breadth among LMNAST *lac* hits (Fig. 3b), and reinforcing the idea of limited *lac* horizontal gene transfer (HGT) [18]. The remainder of the hits consisted of adjacent repurposed characters with functional valence around sugar metabolism.

This survey showed that LMNAST *E. coli lac* operon searches identified numerous ortholog and paralog instances. Relative disparities in gene preservation, gene loss, and structural rearrangements bearing signaling implications were delineated. While there was a significant degree of conformity to the standard genomic arrangement, the amount of diversity indicates that attention paid to related, non-canonical signaling units may be worthwhile.

### *E. coli* K-12 W3110 Lsr System Query

Further testing of LMNAST was conducted with weak, stringent, and expanded window searches of the *E. coli* Lsr system. The query Lsr system consists of a kinase (LsrK: BAA15191), a repressor (LsrR: BAA15192), ABC transporter genes (LsrA: BAA15200, LsrC: BAA15201, LsrD: BAA15202, and LsrB: BAE76456), and phospho-AI-2 (AI-2-P) processing genes (LsrF: BAE76457, LsrG: BAE76458). Along with AI-2, the Lsr system consists of multiple overlapping positive and negative feedback loops. Multimeric LsrR represses system expression emanating from the intergenic region. AI-2-P, itself catabolized by LsrF and LsrG, allosterically relieves that repression. Thus, both expression troughs and peaks are tightly regulated [19]. For the LMNAST search we used the Lsr genes spanning bp 1600331 to 1609003 of *E. coli* K12 substrain W3110 (Genbank nucleotide record AP009048). The number of hits returned using stringent criteria totaled 419.

**Figure 6:**
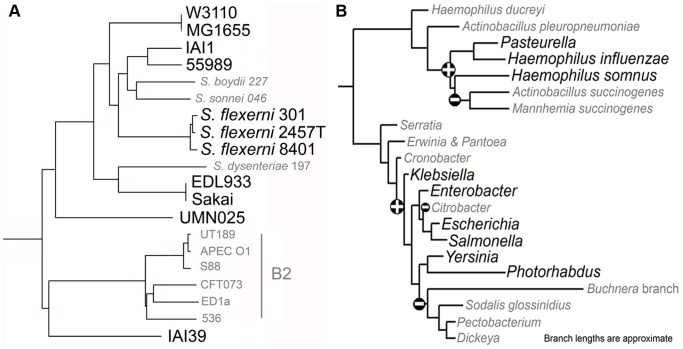
Lsr system LMNAST hits overlaid onto phylogenetic distributions of different scopes. **A**. *E. coli* Lsr system LMNAST hits overlaid onto an *E. coli* specific phylogenetic distribution as developed in and adapted from [4], [27]. The larger, bolded leaves contain Lsr system homologs, whereas the grayed italicized leaves do not. Lsr system loss is evident in B2 strains. **B**. *E. coli* Lsr system LMNAST hits overlaid onto an Enterobacteriales and Pasteurellales phylogenetic distribution adapted from [34]. The larger, bolded leaves contain Lsr system homologs, whereas the grayed italicized leaves do not. Loss and gain events are denoted by – and+respectively based on parsimony. Compared to the distribution of the *lac* operon, Lsr is more dispersed but also shallower, phylogenetically.

Much like the *lac* operon, the Lsr system appeared subject to imperfect conservation. Certainly, many fully sequenced *E. coli* bore exact Lsr homologs (meH>0.95). Exceptions were the truncated systems found in strains BL21 [20], REL606 [20], and E24377A, and the specific and complete excision of Lsr systems from an otherwise preserved gene order in B2 type *E. coli* (Figs. 6 and S1) as revealed through expanded window searching.

**Figure 7:**
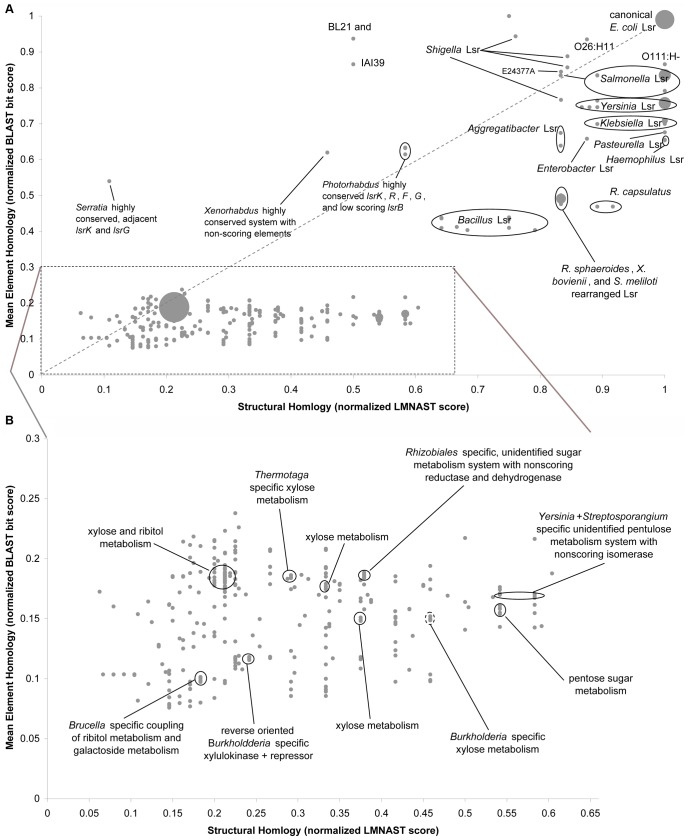
Annotated 2D similarity plot for Lsr system LMNAST weak search hits. A. HGT of homologous systems is evident among hits with perfect organizational homology but diminished mean element homology. A great number of hits have low similarity along both axes. As seen in B., these hits are mostly involved in the metabolism of 5 carbon carbohydrates according to their annotation. This is likely reflective of the fact that AI-2 is of similar structure to 5 carbon sugars.

Unlike the *lac* operon, numerous Lsr system homologs had perfect LMNAST homology but markedly reduced meH (Fig. 7a). This is suggestive of amelioration following recent HGT events (which may itself be a reflection of a carefully tuned signal requiring the full complement and correct arrangement of Lsr elements). Indeed, Lsr system GC content varied in accordance with the background GC content, ranging from 0.35 to 0.71. Finer scale GC analysis revealed a single consistent and curious feature across all hits with meH greater than 0.3: a sharply spiking dip in fractional GC content near the intergenic region (Fig. S2). This dip is suggestive of a conserved DNA binding domain essential to the signal transduction process, which would also, however, be a regulatory feature outside the scope of LMNAST searches.

**Figure 8:**
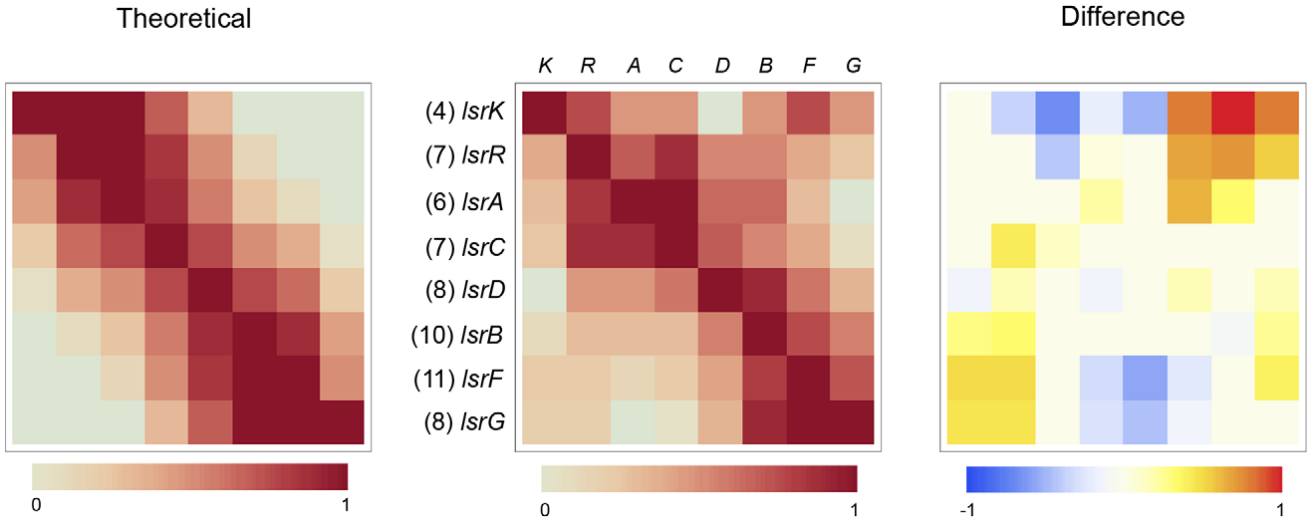
Coincidence matrix for *E. coli* Lsr system LMNAST stringent search hits. This coincidence matrix depicts the subset of hits with a mean element homology >0.3 and also containing 4–6 gene characters. This subset was chosen for its intermediate degree of homology to the query Lsr system. *LsrF* and *lsrG* characters were found to be overrepresented among these hits coincident to hits also containing *lsrR* and l*srK* characters.

Imperfect LMNAST hits with meH greater than 0.3, deviated from the theoretical distribution according to a bias towards the conservation of *lsrB*, *F*, and *G*, relative to the *lsrA*, *C*, and *D* importer genes (Fig. 8). This may be attributable to the fact that *lsrB, F*, and *G* likely pass cell signaling information downstream [14], [21], [22], whereas loss of Lsr importer function might be partially redundant to a low affinity rbs pathway [23], the likely alternate AI-2 import pathway [19], [24], [25].

In contrast to high meH systems, many systems with low meH (<0.3) were involved in the metabolism of 5 carbon sugars, mainly ribitol and xylose, according to Genbank annotation (Fig. 7b). Since AI-2 itself is mainly comprised of a 5 carbon ring, such homology is simultaneously intriguing and unsurprising. More generally among these low similarity hits, *lsrK* characters were commonly coincident with Lsr importer characters (*lsrA*, *C*, *D*, and *B*), indicative of the functional link between such characters. These various features were laid more strongly in relief when measured against the proximal genetic background in an extended window search.

**Figure 9:**
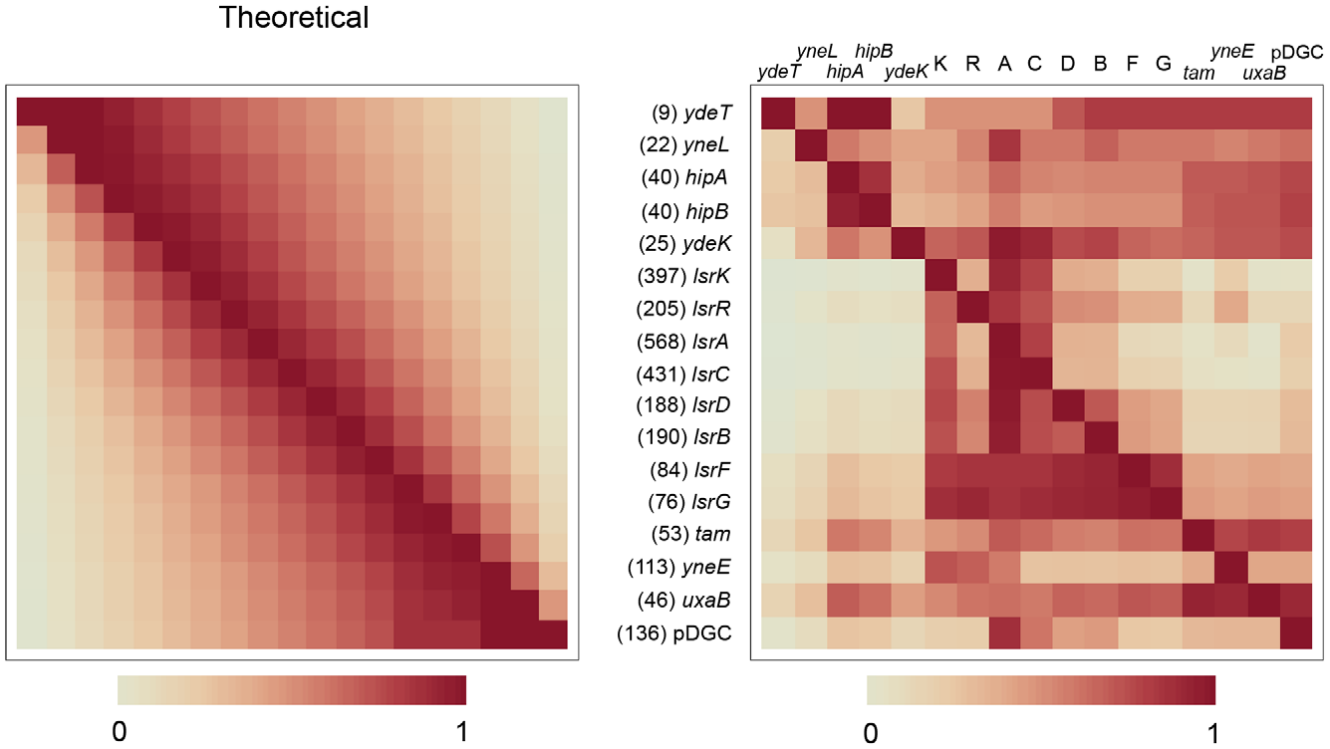
Coincidence matrix for *E. coli* Lsr system LMNAST extended window stringent search hits. Letters represent the respective Lsr genes. 1–5 represent the five genes preceding the Lsr system: *ydeT*, *yneL*, *hipA*, *hipB*, and *ydeK-lipoprotein*. 14–17 represent the four genes after the Lsr system: *tam* (transaconitate methyltransferase), *yneE*, *uxaB*, and a predicted diguanylate cyclase. The figure suggests a) strong conservation of the association between Lsr system genes relative to its neighbors, b) hits in which the Lsr system has been excised entirely from its gene neighbors, and c) a weak coincidence between yneH-glutaminase characters and the Lsr system. The matrix also indicates that the overall prevalence of *lsrF* and *lsrG* characters is lower than other canonical Lsr characters, although the presence of *lsrF* and *lsrG* characters is a good predictor of the presence of other Lsr genes.

While a representation of hit variability preserving structural information can be had from trackback plots (Fig. S1), additional salient results from stringent Lsr extended window searching could be deduced from the more summary coincidence heat maps (Fig. 9). The matrices indicate that *lsrK* and *lsrA* genes were strongly preserved among extended window hits. Also, if either *lsrF* or *lsrG* were present, the remaining Lsr genes were likely present. The complete system rescission mentioned before was hinted at, especially in rows 3 and 4, corresponding to the toxin/antitoxin *hipAB* system. Intra-species variation of structural homology increased greatly when using stringent rather than weak criteria (data not shown), mainly as a result of gene loss to pseudo gene conversion, mostly among transporter genes—a bias most easily explained as a matter of pure probability since there are more transporter genes than any other type, and a fact whose functional significance is blunted by the alternative AI-2 import pathway.

These initial *E. coli* searches motivated other orthologous Lsr system queries. Full results for *E. coli*, *S.* Typhimurium, and *B. cereus* searches are available in Fig. S3. These additional searches helped identify other possible Lsr system homologs, HGT partners, and non-canonical system-associated gene candidates. In Fig. S3, we delineate operon directionality and gene homology. It is interesting to note that system variants exist among noted human pathogens: *Yersinia pestis*, *Bacillus anthracis*, and *Haemophilus influenzae*. In some instances, *lsrRK* are either absent (e.g. *E. coli* BL21) or are associated with altered intergenic regions implying altered regulatory control (e.g. *Yersinia pestis* Antiqua). In other cases transporter genes are distributed with altered bias due to position in the bidirectional operons (e.g. *Yersinia pseudotuberculosis* PB1/+). In some cases there is no *LsrFG* component (e.g. *Shigella flexerni* 2002017). LsrF and LsrG are AI-2-P processing enzymes that lower the intracellular AI-2-P level, thereby contributing to the repression of AI-2 induced genes.

Given even only this modest degree of dispersion, it is nonetheless reasonable to suggest that the Lsr auto-induction system is, in fact, extant among scores of bacterial species and that because the organization of genes within the regulatory architecture is varied, the downstream phenotypic behaviors aligned with AI-2 regulated QS genes is likewise variable. Thus, our results are in line with a general hypothesis that the AI-2 quorum sensing system is broadly distributed and that the specific needs of the bacteria in a given niche are met by disparate operon arrangements.

The overall phylogenetic distribution of the Lsr system mirrors that as developed by Pereira, et al. in the cluster they denote as Group I [26]. Here, however, details were fleshed out with different emphases. The Lsr LMNAST search captured the diversity of pseudo gene conversion, structural rearrangement, and additional hitchhiking genes associated with the Lsr systems that exist in the present nr/nt database. Moreover, inferences regarding regulatory Lsr system signals could be made that might also map to phylogenies or possibly, with much more effort, related ecological niches.

### Analysis of Lsr System Search Results

**Figure 10:**
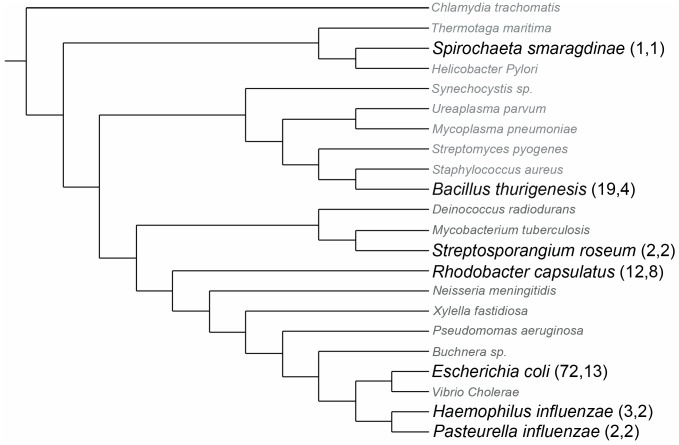
Phylogenetic Distribution of Lsr at different Phylogenetic scales using reconciled LMNAST results. *E. coli* Lsr system LMNAST hits overlaid onto a bacterial phylogenetic distribution as developed in and adapted from [27]. Each leaf bears a representative member from a larger unseen collapsed branch. The larger, bolded leaves contain Lsr system homologs within the collapsed branch, whereas the grayed italicized leaves do not. Parenthesized numbers indicate the number of strains and species with Lsr system LMNAST hits contained within the collapsed branch.

Results from the various LMNAST searches were reconciled by taking the highest scoring hit among overlaps within each nucleotide record. In Fig. 10, we overlaid LMNAST search results onto a phylogenetic tree [27] based on the *E. coli* genome and 16s data. Interestingly, Lsr system homologs clustered mainly in gammaproteobacteria with the greatest density being among *E. coli* strains. Diffusely manifesting in more distantly related bacterial species, the Lsr system appears to have been subject to several HGT events. That is, the Lsr system is absent in numerous Enterobacteriaceae species, while HGT gain events happened at the root of the *Bacillus cereus* group, to *R. sphaeroides* and *R. capsulatus* separately, to *Sinorhizobium meliloti*, and to *Spirochaeta smaragdinae* (Fig. 10). Curiously, while these bacteria occupy distinct ecological niches, they are all common to soil or water environments.

Multiple extended window searches indicated that *S. enterica* was the most proximal cluster for every Lsr system HGT candidate. The sharing of a novel Lsr system-associated “mannose-6-phosphate isomerase” (NP_460428) between *Bacillus cereus* group members, *S. smaragdine*, and *S. enterica*, further strengthened the suggestion of HGT partnership. The gene annotated as “mannose-6-phosphate isomerase” or “sugar phosphate isomerase,” has recently been shown to be part of the LsrR regulon in *Salmonella* [28]. Although not part of the *E. coli* regulon, it was also associated with *S. smaragdinae* and *B. cereus* group orthologs. In keeping with a possible AI-2-P processing role, it was consistently adjacent to *lsrK*.

Among gammaproteobacteria, parsimony suggests that two gain events of the Lsr system occurred: one deeply rooted in enterobacteriales and one in a pasteurellales ancestor. In the enterobacteriales branch, besides *Escherichia*, *Shigella*, and *Salmonella*, Lsr organizational homologs were found in *Enterobacter*, *Photorhabdus*, and *Xenorhadbus* species, although most of these instances lacked importer genes (*lsrACDB*).

While it is thought that regulatory proteins conserved across such long phylogenetic distances often regulate different targets [29], the regulation of community-related functions by different manifestations of the Lsr system (such as biofilm maturation checkpoints in *E. coli* [25] and possible biofilm dispersion in *B. cereus* [12]) suggests a convergent tendency to leverage a quorum/environment sensing capacity inherent to the Lsr system. Indirect influence over a broader regulon may be abetted by the involvement of AI-2, the Lsr system substrate, in metabolic pathways [30].

#### Putative Lsr system in Rhizobiales

A grouping of Rhizobiales common to plant symbioses had a conserved set of adjacent, low homology elements and nonscoring elements that consisted of unidirectionally expressed genes annotated as: ABC-type transporters (ribose, putative, putative), a DeoR family repressor (LsrR synonym), aldo-keto reductase (non-scoring), glycerol-3-phosphate dehydrogenase (nonscoring LsrF synonym), fructose-biphosphate aldolase (LsrF synonym), and xylulose kinase (LsrK synonym). Somewhat inescapably, synonymy is at least partly a function of precedence resulting in attribution bias. The indeterminacy of signaling similarity between these homologs and better characterized Lsr systems suggests a need for further research.

## Discussion

LMNAST is a program that evaluates similarity or homology on the level of gene organization, conducting a search for patterns and prevalence constrained by a BLAST E-value filter. Program results overlaid onto phylogenetic data allow visual inspection of phylogenetic density and dispersion. 2D homology plots display system variability among LMNAST orthologs, and when overlaid with genera/species clustering, reveal the degree of system conservation within and across genera/species when organizational homology decreases and element homology is constant. Clustering also enables the identification of conserved system homologs. Organizational information is lost when using coincidence heat charts, but suggestions of the underlying structural variability remain nonetheless. This is particularly true for coincidence representations of extended window searches. For such searches, contextual associations with non-canonical genes may also emerge. Trackback plots illustrate both variety and structural information, albeit in a less dense format. These representations are especially useful in combination. It should be noted that the results are almost entirely comprised of excerpts from fully sequenced genomes. Results are also biased by BLAST input, as characters with more element homologs (e.g. *lsrA*) appear more frequently in hits.

Generically, LMNAST identified query homologs with a variety of deletions, insertions, misordering, and misdirections. While nearly any source of mutagenesis may result in a frank mutation affecting a system's organizational homology, homologous recombination, insertion sequences, transposable elements, and combinations thereof are likely to be of particular consequence for LMNAST searches. Deletions may be a result of pseudo gene conversion, of chromosomal rearrangements, or part and parcel of an insertion event—if the insertion results in a gap sufficiently large as to disconnect hit elements from one another. In the case of such insertions, sufficiently weak criteria may be of use, with the caveat that decreased stringency increases the number of false positives. From a signaling perspective, depending on the impacted elements and the nature of the inserted sequence, gap presence could result in system discoordination; and the longer the gap the more probable and severe the discoordination, most likely to the detriment of system function.

As for the specific test queries examined herein, while the *lac* operon is well characterized in its canonical form, there nonetheless exists a great deal of frank variation from the textbook case. Of particular interest were homologous instances where structural rearrangement could influence self-regulation of component expression. Also of note were its multiple signaling component deletions. Such abbreviated homologs were frequently repurposed in a related context. Complete *lac* operons were found among nearly all *E. coli* strains. Incomplete *lac* operons were found to be distributed only among closely related Enterobacteriaceae species comprised almost entirely of *Escherichia*, *Citrobacters*, *Enterobacters*, and *Serratias* as expected based on limited *lac* operon HGT [18]. This difference between the rates of decay for the two homology signals over phylogenetic space may be suggestive of distinct selection pressures guiding the two systems. Also identified through LMNAST were conserved, *E. coli*-specific evolved beta-galactosidase systems [17], demonstrating a capacity to find directly evolved but highly distinct (meH∼0.19) homologs.

On par, Lsr system hit structural similarity was less well correlated with meH than *lac* operon results, a phenomenon presumably associated with apparent Lsr system HGT. The Lsr system was phylogenetically dispersed more widely than the *lac* operon, even while its distribution remained densest among gammaproteobacteria. Much like the *lac* operon, Lsr system structure was subject to significant variability. *lsrK* and *lsrR* characters were common to many hits. *lsrF* and *lsrG* were the least common; the inclusion of both elements nearly always coincided with the presence of all other Lsr characters as well. Lsr-contextually associated genes and novel putative Lsr systems were also elucidated.

The dispersion of Lsr to bacteria as far afield as the *S. smaragdinae*, the first *Spirochaeta* to be fully sequenced [31], is intriguing. It suggests that while the depth of Lsr dispersion may not be significant, that its exposed breadth will expand incrementally at a rate proportional to microbial genome sequencing. While the direct regulon of such HGT systems is expected to be limited [29], [32], the proximity of the substrate to key metabolic pathways may allow the Lsr system to confer contextual phenotypic advantages by impacting downstream pathways with its capacity to recompartmentalize a metabolic intermediate. Moreover, the known regulatory requirements for functional integration of the Lsr system are minimal, consisting entirely of interaction with cAMP-CRP complex, which is deeply rooted in eubacteria. Gene organization differences between dispersed Lsr homologs, may indicate distinct signaling outcomes, in turn suggesting the appropriation of the Lsr system's inherent quorum capacity to drive distinct phenotypes suited to a given bacteria's needs within its particular niche.

Unlike the results for the *lac* operon, Lsr system results returned a large number of other-annotated, low homology systems. This speaks to both the inherent difficulty of extrapolation based on homology and the utility of the additional, complementary homology measure yielded by LMNAST searching. Overall, given the complexity of the results, numerous aspects may be of interest. For example, extant variation of the queried modular systems, as captured by frank changes in gene organization, was revealed. Several topological curiosities were also revealed. For example, the Lsr system's apparent dispersion through both horizontal and vertical inheritance could, in fact, suggest that quorum sensing behavior that is regulated by the Lsr system is conveyed as a root of selective advantage, as opposed to the specific regulon known to uptake small molecules that could otherwise be viewed as carbon source. By considering our results in the context of common graphical tools of a complementary nature (e.g. 2D similarity plots and coincidence heat maps), through LMNAST we offer a new avenue by which to explore this and other provocative questions.

## Supporting Information

Figure S1 **Trackback plots for Lsr system LMNAST extended window stringent search hits.** These diagrams describe the variety of LMNAST hits in greater detail. A straight diagonal line indicates complete agreement with the query. Rearrangements are represented by discontinuities. Relative redirection is indicated by a flipping of the diagonal orientation. Deletion is indicated by horizontal dashed gaps. Insertion is indicated by vertical gaps. The legend in the upper right hand corner indicates which numbers correspond to which genes. Trackback plots are organized into categories: A, B, C, D, and E according to the following: I. Prototype Lsr systems, II. Modified Lsr systems with pre and post-Lsr adjacent characters, III. Modified Lsr systems with post-Lsr adjacent characters, IV. Modified systems without Lsr adjacent characters, and V. Highly modified Lsr systems. For exact subgroup membership see Table S1.
(TIF)

Figure S2 **GC content demonstrates consistent spiking dip at intergenic region.** GC content graphs for *Bacillus cereus* ATCC10967, *Yersinia Pestis* Nepal516, *Rhodobacter sphaeroides* ATCC 17029, and *Yersinia pestis* CO92. Graphs are labeled with Lsr system beginning (end of *lsrK*), *lsrR* gene intersection with the intergenic region (*lsrR* end), and Lsr system ending (end of *lsrG*). Arrow direction indicates the direction of *lsrACDBFG* expression.
(TIF)

Figure S3 **Merged results from three separate LMNAST searches for Lsr system homologs.** The *E. coli* K-12 W3110 search shown was completed using weak criteria (a lower gap extension penalty and less rigid adherence to annotation), whereas the *B. Cereus* ATCC 10987 and *S. enterica* typhimurium LT2 searches used stringent criteria (higher penalties for deviation from the query pattern and adherence to supplied annotation). “Join” indicates a difficulty in handling uncommon annotation where the first gene annotated in a record spans the end and the beginning of the record. Arrow direction indicates the direction of transcription along the genome. Color is a stand-in for character type, and the degree of shading indicates degree of element homology, with the darkest shade representing 100% element homology.
(TIF)

Table S1 **Trackback plot subcategories for Figure S1.** Species represented in the trackback plot from an *E. coli* K-12 W3110 Lsr system expanded window stringent search.
(DOC)
