# Supplementary figures and images for: Gene Network Homology in Prokaryotes Using a Similarity Search Approach: Queries of Quorum Sensing Signal Transduction
Source: PLoS Comput Biol. 2012 Aug 16;8(8):e1002637. doi: 10.1371/journal.pcbi.1002637 (PMC3420918; doi:10.1371/journal.pcbi.1002637)

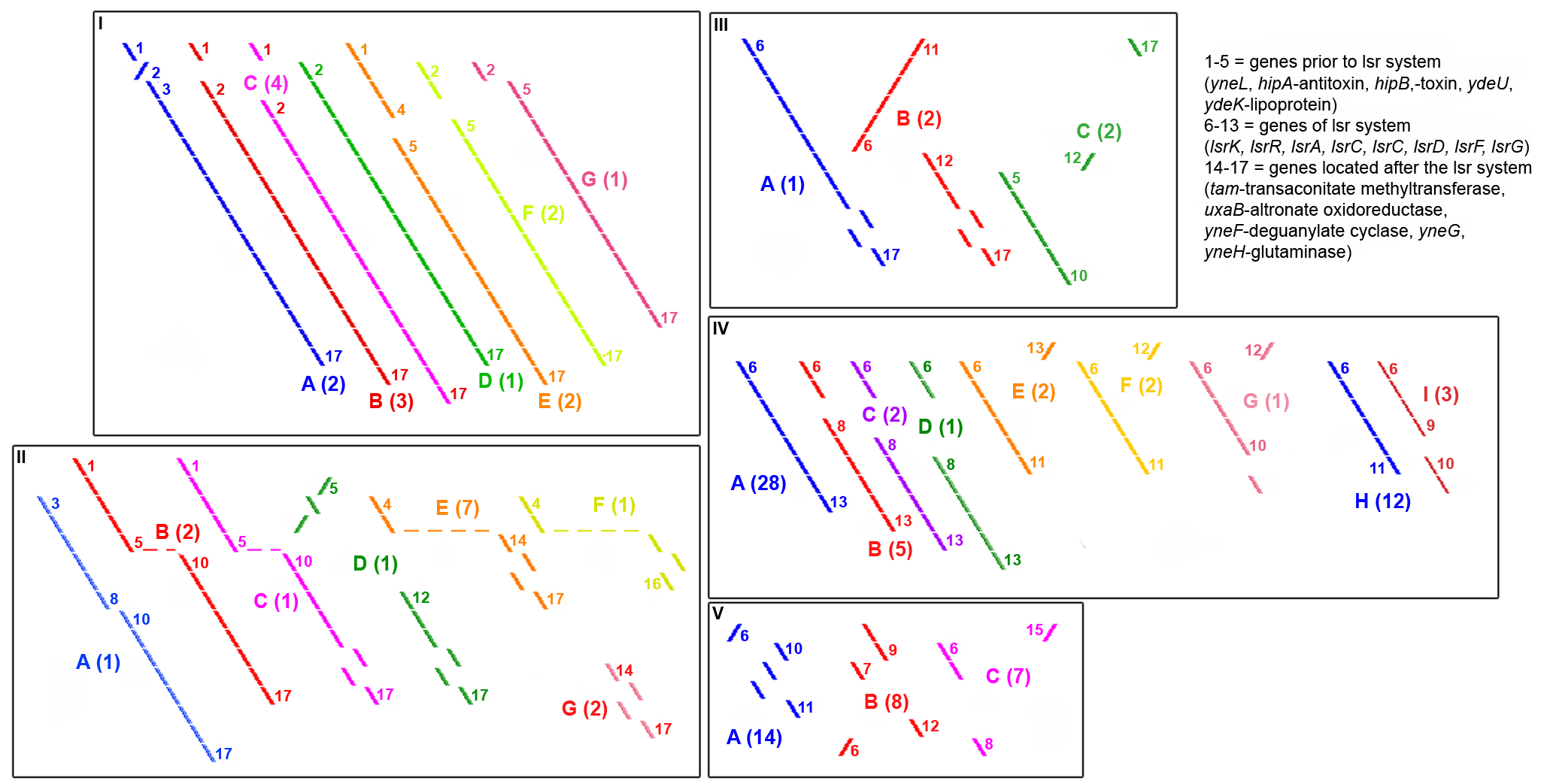

Supplement: Figure S1 — Trackback plots for Lsr system LMNAST extended window stringent search hits. These diagrams describe the variety of LMNAST hits in greater detail. A straight diagonal line indicates complete agreement with the query. Rearrangements are represented by discontinuities. Relative redirection is indicated by a flipping of the diagonal orientation. Deletion is indicated by horizontal dashed gaps. Insertion is indicated by vertical gaps. The legend in the upper right hand corner indicates which numbers correspond to which genes. Trackback plots are organized into categories: A, B, C, D, and E according to the following: I. Prototype Lsr systems, II. Modified Lsr systems with pre and post-Lsr adjacent characters, III. Modified Lsr systems with post-Lsr adjacent characters, IV. Modified systems without Lsr adjacent characters, and V. Highly modified Lsr systems. For exact subgroup membership see Table S1. (TIF) [file pcbi.1002637.s001.tif]

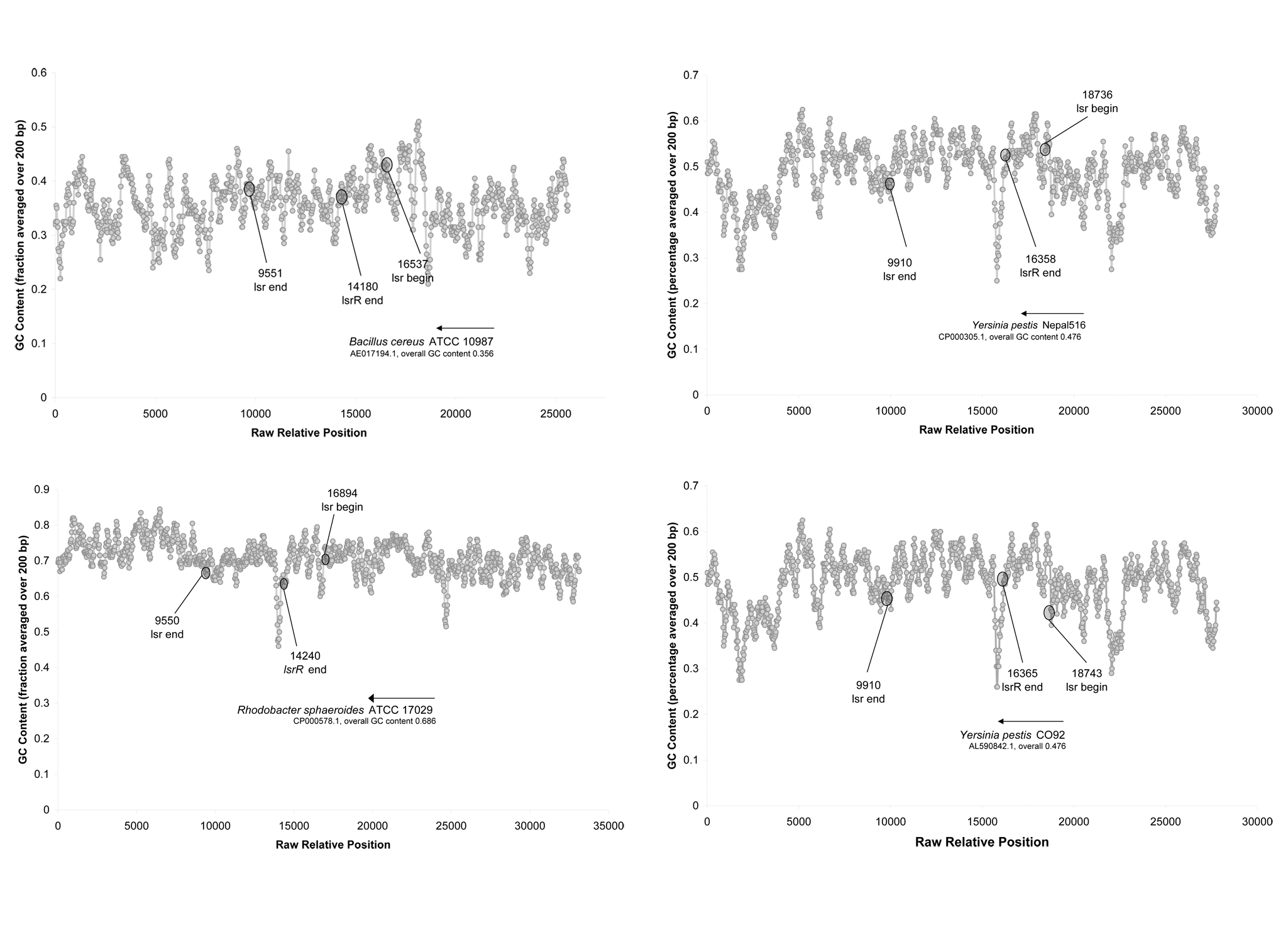

Supplement: Figure S2 — GC content demonstrates consistent spiking dip at intergenic region. GC content graphs for Bacillus cereus ATCC10967, Yersinia Pestis Nepal516, Rhodobacter sphaeroides ATCC 17029, and Yersinia pestis CO92. Graphs are labeled with Lsr system beginning (end of lsrK), lsrR gene intersection with the intergenic region (lsrR end), and Lsr system ending (end of lsrG). Arrow direction indicates the direction of lsrACDBFG expression. (TIF) [file pcbi.1002637.s002.tif]

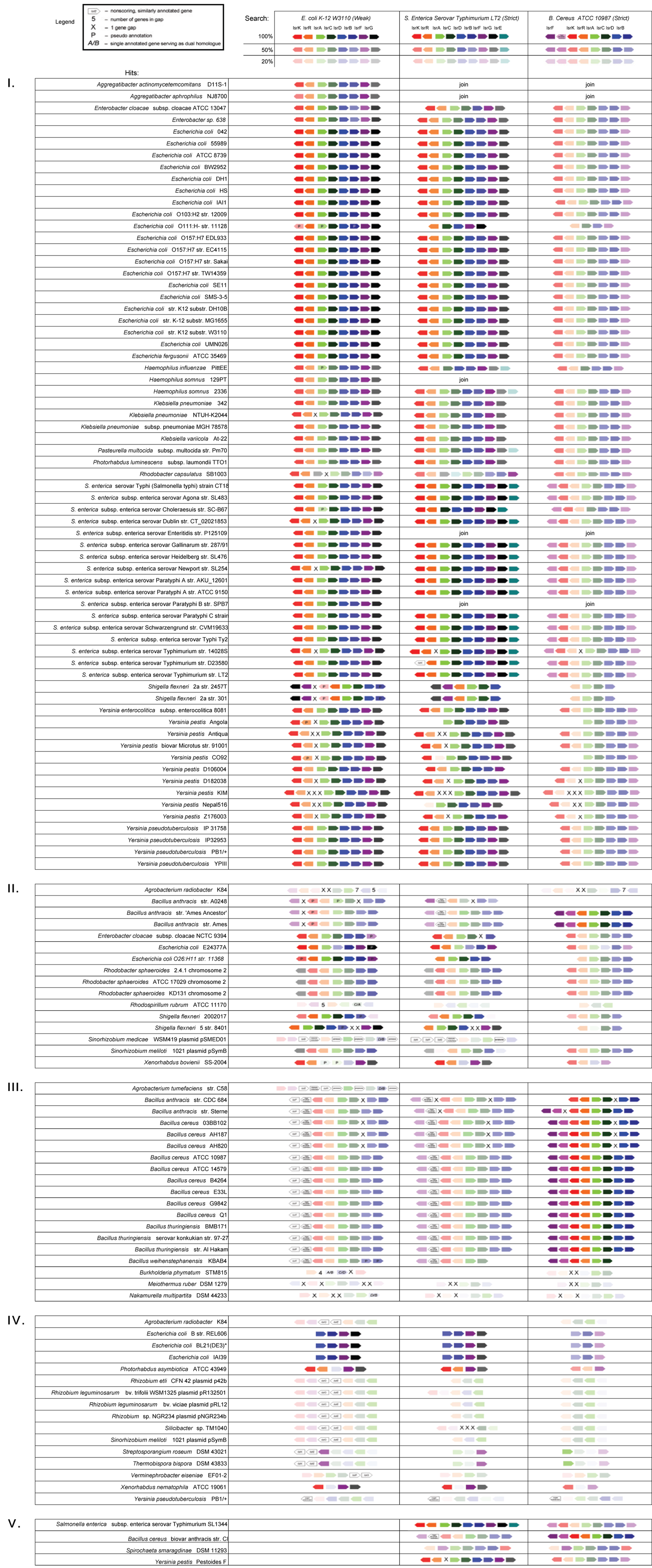

Supplement: Figure S3 — Merged results from three separate LMNAST searches for Lsr system homologs. The E. coli K-12 W3110 search shown was completed using weak criteria (a lower gap extension penalty and less rigid adherence to annotation), whereas the B. Cereus ATCC 10987 and S. enterica typhimurium LT2 searches used stringent criteria (higher penalties for deviation from the query pattern and adherence to supplied annotation). “Join” indicates a difficulty in handling uncommon annotation where the first gene annotated in a record spans the end and the beginning of the record. Arrow direction indicates the direction of transcription along the genome. Color is a stand-in for character type, and the degree of shading indicates degree of element homology, with the darkest shade representing 100% element homology. (TIF) [file pcbi.1002637.s003.tif]
