# Supplementary material for: Gene Network Homology in Prokaryotes Using a Similarity Search Approach: Queries of Quorum Sensing Signal Transduction
Source: PLoS Comput Biol. 2012 Aug 16;8(8):e1002637. doi: 10.1371/journal.pcbi.1002637 (PMC3420918; doi:10.1371/journal.pcbi.1002637)
Supplement: Table S1 — Trackback plot subcategories for Figure S1. Species represented in the trackback plot from an E. coli K-12 W3110 Lsr system expanded window stringent search. (DOC) [file pcbi.1002637.s004.doc]

Table S1

| Group | Subgroup | Species-Strain |
| --- | --- | --- |
| I (pre-Lsr-post) | A | *Escherichia coli* ATCC 8739 |
|  |  | *Escherichia coli* DH1 |
|  | B | *Escherichia coli* O103:H2 str. 12009 |
|  |  | *Escherichia coli* O157:H7 str. EC4115 |
|  |  | *Escherichia coli* SE11 |
|  | C | *Escherichia coli* O111:H- str. 11128 |
|  |  | *Escherichia coli* O157:H7 str. TW14359 |
|  |  | *Escherichia coli* O26:H11 str. 11368 |
|  |  | *Escherichia coli* 55989 chromosome |
|  | D | *Escherichia coli* IAI1 |
|  | E | *Escherichia coli BW2952* |
|  |  | *Escherichia coli str. K12 substr. DH10B* |
|  | F | *Escherichia coli* O157:H7 str. Sakai |
|  |  | *Escherichia coli* O157:H7 EDL933 |
|  | G | *Escherichia coli* SMS-3-5 |
| II (pre-modified Lsr-post) | A | *Escherichia coli* E24377A |
|  | B | *Escherichia coli* B str. REL606 |
|  |  | *Escherichia coli* BL21(DE3) |
|  | C | *Escherichia coli* IAI39 |
|  | D | *Shigella dysenteriae* Sd197 |
|  | E | *Escherichia coli* 536 |
|  |  | *Escherichia coli* APEC O1 |
|  |  | *Escherichia coli* CFT073 |
|  |  | *Escherichia coli* IHE3034 |
|  |  | *Escherichia coli* S88 chromosome |
|  |  | *Escherichia coli* SE15 |
|  |  | *Escherichia coli* UTI89 |
|  | F | *Escherichia coli* ED1a chromosome |
|  | G | *Escherichia coli* 042 |
|  |  | *Shigella boydii* CDC 3083-94 |
| III (modified Lsr-post) | A | *Escherichia fergusonii* ATCC 35469 |
|  | B | *Shigella flexneri* 2a str. 2457T |
|  |  | *Shigella flexneri* 2a str. 301 |
|  | C | *Sinorhizobium meliloti* 1021 plasmid pSymB |
|  |  | *Rhodobacter sphaeroides* KD131 chromosome 2 |
| IV (modified Lsr) | A | *Aggregatibacter aphrophilus* NJ8700, complete genome |
|  |  | *Enterobacter* sp. 638 |
|  |  | *Haemophilus influenzae* PittEE |
|  |  | *Haemophilus somnus* 129PT |
|  |  | *Haemophilus somnus* 2336 |
|  |  | *Klebsiella pneumoniae* 342 |
|  |  | *Klebsiella pneumoniae* subsp. pneumoniae MGH 78578 |
|  |  | *Pasteurella multocida* subsp. multocida str. Pm70 |
|  |  | *Salmonella enterica* subsp. enterica serovar Agona str. SL483 |
|  |  | *Salmonella enterica* subsp. enterica serovar Choleraesuis str. SC-B67 |
|  |  | *Salmonella enterica subsp.* enterica serovar Enteritidis str. P125109 |
|  |  | *Salmonella enterica* subsp. enterica serovar Gallinarum str. 287/91 |
|  |  | *Salmonella enterica* subsp. enterica serovar Heidelberg str. SL476 |
|  |  | *Salmonella enterica* subsp. enterica serovar Paratyphi A str. AKU_12601 |
|  |  | *Salmonella enterica* subsp. enterica serovar Paratyphi A str. ATCC 9150 |
|  |  | *Salmonella enterica* subsp. enterica serovar Paratyphi B str. SPB7 |
|  |  | *Salmonella enterica* subsp. enterica serovar Paratyphi C str. RKS 4594 |
|  |  | *Salmonella enterica* subsp. enterica serovar Schwarzengrund str. CVM19633 |
|  |  | *Salmonella enterica* subsp. enterica serovar Typhi Ty2 |
|  |  | *Salmonella enterica* subsp. enterica serovar Typhimurium str. D23580 |
|  |  | *Salmonella enterica* subsp. enterica serovar Typhimurium str. LT2 |
|  |  | *Yersinia enterocolitica* subsp. enterocolitica 8081 |
|  |  | *Yersinia pestis* Angola |
|  |  | *Yersinia pestis* CO92 |
|  |  | *Yersinia pseudotuberculosis* IP 31758 |
|  |  | *Yersinia pseudotuberculosis* IP32953 |
|  |  | *Yersinia pseudotuberculosis* PB1/+ |
|  |  | *Yersinia pseudotuberculosis* YPIII |
|  | B | *Klebsiella pneumoniae* NTUH-K2044 DNA |
|  |  | *Salmonella enterica* subsp. enterica serovar Dublin str. CT_02021853 |
|  |  | *Salmonella enterica* subsp. enterica serovar Newport str. SL254 |
|  |  | *Salmonella enterica* subsp. enterica serovar Typhimurium str. 14082S |
|  |  | *Yersinia pestis* biovar Microtus str. 91001 |
|  |  | *Yersinia pestis* Pestoides F |
|  | C | *Yersinia pestis* Antiqua |
|  |  | *Yersinia pestis* Nepal516 |
|  | D | *Yersinia pestis* KIM |
|  | E | *Rhodobacter sphaeroides* 2.4.1 chromosome 2 |
|  |  | *Rhodobacter sphaeroides* ATCC 17029 chromosome 2 |
|  | F | *Bacillus anthracis* str. ‘Ames Ancestor’ |
|  |  | *Bacillus anthracis* str. Ames |
|  | G | *Bacillus anthracis* str. A0248 |
|  | H | *Bacillus anthracis* str. CDC 684 |
|  |  | *Bacillus anthracis* str. Sterne |
|  |  | *Bacillus cereus* ATCC 10987 |
|  |  | *Bacillus cereus* ATCC 14579 |
|  |  | *Bacillus cereus* B4264 |
|  |  | *Bacillus cereus* E33L |
|  |  | *Bacillus cereus* G9842 |
|  |  | *Bacillus cereus* Q1 |
|  |  | *Bacillus thuringiensis* serovar konkukian str. 97-27 |
|  |  | *Bacillus thuringiensis* str. Al Hakam |
|  |  | *Bacillus weihenstephanensis* KBAB4 |
|  |  | *Shigella sonnei* Ss046 |
|  | I | *Bacillus cereus* AH187 |
|  |  | *Bacillus cereus* AH820 |
|  |  | *Bacillus cereus* 03BB102 |
| V (Non-continous) | A | *Escherichia coli* ED1a chromosome |
|  |  | *Serratia proteamaculans* 568 |
|  |  | *Shewanella halifaxensis* HAW-EB4 |
|  |  | *Yersinia pestis* Angola |
|  |  | *Yersinia pestis* Antiqua |
|  |  | *Yersinia pestis* biovar Microtus str. 91001 |
|  |  | *Yersinia pestis* CO92 |
|  |  | *Yersinia pestis* KIM |
|  |  | *Yersinia pestis* Nepal516 |
|  |  | *Yersinia pestis* Pestoides F |
|  |  | *Yersinia pseudotuberculosis* IP31758 |
|  |  | *Yersinia pseudotuberculosis* IP32953 |
|  |  | *Yersinia pseudotuberculosis* PB1/+ |
|  |  | *Yersinia pseudotuberculosis* YPIII |
|  | B | *Agrobacterium radiobacter* K84 chromosome 2 |
|  |  | *Ochrobactrum anthropi* ATCC 49188 chromosome 2 |
|  |  | *Rhizobium leguminosarum* bv. trifolii WSM1325 plasmid pR132501 |
|  |  | *Rhizobium leguminosarum* bv. viciae plasmid pRL12 |
|  |  | *Rhizobium sp.* NGR234 plasmid pNGR234b |
|  |  | *Silicibacter sp.* TM1040 |
|  |  | *Sinorhizobium medicae* WSM419 plasmid pSMED01 |
|  |  | *Sinorhizobium meliloti* 1021 plasmid pSymB |
|  | C | *Burkholderia cenocepacia* AU 1054 chromosome 1 |
|  |  | *Burkholderia cenocepacia* HI2424 chromosome 1 |
|  |  | *Burkholderia cenocepacia* J2315 chromosome 1 |
|  |  | *Burkholderia cenocepacia* MC0-3 chromosome 1 |
|  |  | *Burkholderia multivorans* ATCC 17616 DNA |
|  |  | *Burkholderia sp.* 383 chromosome 1 |
|  |  | *Burkholderia xenovorans* LB400 chromosome 1 |
